# Linking Dispense Data to Electronic Health Orders: Tutorial for Querying Commercial Pharmacy Databases to Support Systemwide Quality Improvement

**DOI:** 10.2196/90954

**Published:** 2026-10-06

**Authors:** Benjamin Michaels, Jessica Pourian

**Affiliations:** 1 Department of Pharmacy, University of California, San Francisco, 1500 Owens Street, San Francisco, CA, 94158, United States, 1 513 658 0104; 2 Division of Clinical Informatics and Digital Transformation, Department of Pediatrics, University of California, San Francisco, San Francisco, CA, United States

**Keywords:** electronic health record, pharmacy information systems, quality improvement, clinical informatics, health information exchange, drug prescriptions

## Abstract

**Background:**

Assessing medication adherence is central to quality care, yet linking electronic health record (EHR) medication orders to outpatient pharmacy dispense data remains technically complex.

**Objective:**

This study aimed to present a generalized, reproducible tutorial for linking EHR medication orders to pharmacy dispense data that can be used to assess medication dispense proportions.

**Methods:**

We developed and validated a structured query approach to link EHR medication orders to external pharmacy dispense data using patient identifiers, medication-level identifiers, pharmacy identifiers, and temporal constraints. The tutorial emphasizes key design decisions, including handling multiple triggering events, deduplication across vendors, and managing formulation changes. A retrospective cohort of pediatric acute otitis media encounters (January 1, 2021, to January 1, 2024) was used as an illustrative example.

**Results:**

Overall, 98.3% (302/307) of pharmacies in the cohort returned at least 1 dispense record during the study period and were therefore classified as reporting pharmacies. Among 3404 orders, 2616 (76.9%) had a recorded dispense.

**Conclusions:**

EHR-integrated pharmacy data provide a feasible, timely proxy for assessing medication adherence. This tutorial provides a scalable framework for linking EHR and pharmacy data for medication adherence studies, while highlighting key methodological considerations for SQL coding.

## Introduction

Medication adherence is a critical determinant of clinical outcomes. Understanding and quantifying medication use allow clinicians to tailor care, as nonadherence has been associated with increased health care use and morbidity [1].

Medication adherence is often evaluated using patient surveys, which can be cumbersome, prone to recall bias, and limited in their ability to capture objective data [1,2]. Prescription claims data provide a validated method for assessing medication adherence, including calculation of the proportion of days covered, but may be subject to delays and may exclude patients who are uninsured or who pay out of pocket [2-4].

An alternative is to use noninstitutional medication dispense data, which are often extracted, transformed, and loaded into electronic health records (EHRs) via third-party vendors [5]. Two of the most common third-party vendors are DrFirst [6] and Surescripts [7]. The data from these vendors have discrete elements that can be used to link back to the EHR medication order [8]. Using these resources allows for near real-time access to dispense data. By leveraging commercial pharmacy databases, dispense data can also be matched one-to-one with medication orders, enabling large-scale studies of medication adherence patterns across populations. Previous research has demonstrated that these data capture medication dispenses for the majority of patients and may be used for quality improvement (QI) projects assessing adherence [2,5,9,10]. This methodology is particularly valuable for epidemiologic studies because it directly links clinical encounters to prescription dispenses, allowing differentiation between true medication nonadherence and cases in which a prescription was not renewed—an important limitation of claims-based data [4,11]. Importantly, these data include patients without insurance or those who may pay out of pocket—the dispense is recorded in these databases if the pharmacy dispenses the medication, regardless of the payment method.

We began exploring this method during a QI project at our institution examining the use of watch-and-wait (also known as “delayed”) antibiotic prescribing for pediatric acute otitis media (AOM) in the outpatient setting. Delayed prescribing—referred to as a safety net antibiotic prescription (SNAP) at our institution—is challenging to study because claims data and patient surveys alone cannot reliably determine whether or when a prescribed SNAP is dispensed. To address this, we linked clinical encounters to pharmacy dispense data to assess the time to dispense from the encounter.

In this tutorial, we present a generalizable approach for linking EHR prescribing data with pharmacy dispense data, with a focus on reproducible, SQL-based data engineering methods that can be adapted across institutions regardless of the EHR system. A separate component of our prior work used a large language model to distinguish SNAPs from antibiotics intended for immediate use; that work has been published elsewhere and is not covered here [12,13]. For the purposes of this tutorial, we focused on the antibiotics that were meant to start immediately (not SNAPs), as they were intended to be dispensed and used on the day of the encounter, and thus have higher dispense proportions than SNAPs and should be more in line with other, more typically used medications that the reader may be interested in evaluating. For these reasons, we believe these prescriptions provide a better illustrative example for the SQL methods described herein and for what the reader may anticipate in terms of dispense rates for their own project.

## Tutorial

This section is structured as a step-by-step guide to linking EHR medication orders with external pharmacy dispense data, using a retrospective study of pediatric antibiotics for AOM as an illustrative case.

### Step 1: Define the Study Population and Unit of Analysis

#### Case Study: Pediatric AOM Cohort

Before linking the orders to the dispense history, you must consider the granularity of the data that you are starting with alongside the goal. For this example, the unit is the medication order, not the patient. This allows for the tracking of individual prescription lifecycles from order to fulfillment. Depending on your individual use case, you may want the data to be pulled and aggregated at the patient, department, facility, or another level.

For our use case, we did not assess medication dispense at the patient level across multiple encounters, though this is certainly an option for future studies.

#### Case Study Example

We included outpatient and emergency department encounters for pediatric patients (aged 6 mo to <18 y) diagnosed with AOM using *International Classification of Diseases*, Tenth Revision, Clinical Modification (ICD-10) codes H65, H66, and H67 [14,15], between January 1, 2021, and January 1, 2024. The final cohort consisted of 3404 antibiotic orders for amoxicillin, amoxicillin-clavulanate, or cefdinir. An attrition diagram describing the encounter selection is in Multimedia Appendix 1.

The included 3404 encounters were distributed among 2930 unique patients. Of these patients, 368 had multiple encounters, contributing a total of 842 encounters, with 2 to 6 encounters per patient (Multimedia Appendix 2). A description of the demographics for these 3404 encounters is provided in Table 1.

Because this study was restricted to children in California, where pediatric health coverage is available through Medi-Cal regardless of their parents’ employment or immigration status, nearly all patients in the cohort were insured through either public or private insurance.

**Table 1.** Encounter population (N=3404)^a^.

Category	Overall, n (%)
Sex	
Female	1498 (44.0)
Male	1906 (56.0)
Race	
Asian	397 (11.7)
Black	535 (15.7)
Declined/unknown	66 (1.9)
Mixed	299 (8.8)
Native	21 (0.6)
Other	1436 (42.2)
White	650 (19.1)
Clinic type	
Emergency room	2015 (59.2)
Outpatient	1389 (40.8)
Language	
English	2547 (74.8)
Other	857 (25.2)
Age, y	
6 mo to <1 y	756 (22.2)
1 to <1.5	373 (11.0)
1.5 to <2	234 (6.9)
2 to <5	1127 (33.1)
5 to <10	690 (20.3)
10 to <18	224 (6.6)
Insurance type	
Private	1018 (29.9)
Public	2347 (68.9)
Self-pay	39 (1.1)
Prescribed antibiotic	
Amoxicillin	2757 (81.0)
Amoxicillin-clavulanate	544 (16.0)
Cefdinir	103 (3.0)
Ethnicity	
Declined	66 (1.9)
Hispanic or Latino	1416 (41.6)
Not Hispanic or Latino	1918 (56.3)
Unknown	4 (0.1)

a Patients may have had more than one encounter if they presented more than once in the study period.

### Step 2: Define Key Concepts and Data Elements

#### Case Study: EHR Data Retrieval Settings

For the second step, there are a few baseline definitions that will be used to describe the process of pulling this data:

- Foreign key: a field in SQL databases used to link records across relational tables (eg, patient ID)
- Triggering event: an EHR interaction (eg, an appointment, refill request, or telephone encounter) that initiates the batch retrieval of external pharmacy data
- Match proportion: the percentage of orders successfully linked to a dispense record

In this step, it is important to define these elements and to be aware of what the triggering event settings are within your EHR. A triggering event activates the pulling of data from the external dispense data vendor for each event, and the data pull is often marked with the date when the data were retrieved. It is also important to be aware of the look-back period for the data pull.

#### Case Study Example

At our institution, the University of California, San Francisco (UCSF), the system retrieves up to 365 days of prior dispense data at the time of a triggering event. Triggering events in our EHR include refill encounters, order encounters, hospital encounters, appointments, patient messages, or telephone encounters.

Within our system, there is no distinct foreign key that can be used to match the data to the external dispense data. The order ID for a specific medication in our system is not reflected in the commercial database. Thus, we cannot easily match an order from an encounter to a specific dispense from the pharmacy database. As a result, we had to develop surrogate key logic with multiple steps, which is outlined in Step 4.

### Step 3: Data Extraction

#### Case Study: EHR Data Extraction

Data should be extracted from the EHR reporting database. This typically involves developing and testing a SQL query. The key step here is to identify the order data, patient information, and pharmacy and medication information. Researchers may consider including the following to augment their analysis:

- Encounter data: clinic setting, department, date, time of appointment or encounter, time that the encounter was made, check-in time, diagnosis code, encounter note, and patient after-visit summary
- Order data: order time, order date, order number, and ordering provider
- Patient information: name, patient identifier, birthdate, address, sex, race, ethnicity, language, and insurance status
- Pharmacy: pharmacy name, phone number, and address
- Medication information: name, concentration, dose, and comments to the pharmacy

Next, it is important to locate the external dispense data and, if necessary, join it to the same patient, pharmacy, and medication information tables.

#### Case Study Example

Data were extracted from Epic Clarity. The SQL queries used for data extraction were developed and internally reviewed by the study authors. The original code was written by JP and subsequently updated and revised by BM. No separate IT or external technical review of the SQL code was performed. Both authors are Epic Clarity–certified.

The research team performed random sampling of records to verify data integrity and ensure inclusion criteria were met. We were able to identify the order, patient, medication, and pharmacy tables, along with the external dispense data tables. Due to copyright restrictions and privacy agreements governing the proprietary EHR data tables, we are unable to share the exact code used in this project. Instead, we provide a simplified, illustrative outline of the coding approach on GitHub [16], and a Jupyter notebook containing deidentified practice data is also provided on GitHub [17]. An archived version corresponding to this publication is available through Zenodo [18]. The repository is intended to demonstrate the general analytic workflow and is not a runnable or complete implementation of the analysis; therefore, it should not be used to reproduce the analysis directly.

Our institution has a proprietary agreement with DrFirst for the majority of our database dispenses. We also maintain a relationship with Surescripts as a backup and for certain insurance types.

### Step 4: Linkage Strategy

#### Case Study: EHR and External Dispense Data Linkage

Your EHR may not contain a foreign key that can be used to link the external dispense information to the internal order (such as an order ID). To match at the order level, this can be accomplished by leveraging surrogate secondary keys in a multilookup configuration to ensure you have the correct order. The patient, medication, and pharmacy identifiers are often present in both the ordering and the outpatient dispense tables within the database. All three should be used at a minimum. Additionally, if needed for the use case, ordering provider identifiers, such as a Drug Enforcement Administration number or a National Provider Identifier (NPI), can be leveraged. The reader should note that clinics with multiple ordering providers—trainees, such as residents and fellows, or advanced practice providers—may have discrepancies between the ordering provider and the attending physician who signed the visit.

#### Case Study Example

Linking the EHR encounter to the correct outpatient dispense record was the central challenge of our project. The foreign key that our EHR vendor suggested would be associated with the order data was unusable because the order number sent by our EHR does not match the prescription number used by the pharmacy processing software. Additionally, the dispense data received did not contain the original EHR order number that we could locate.

Identification of a common key is a challenge that most EHRs face with outpatient, external prescription dispense data, as internal data primary keys rarely match those used by external pharmacies or databases. Thus, multiple matches using secondary keys must be used with conditional join logic to establish the correct link. This is in contrast to internal inpatient hospital pharmacies, which will likely share order IDs, thereby simplifying the linkage between encounters and dispenses.

For our outpatient use case, we used Generic Control Number (GCN; First DataBank [19])–based matching to group across brand/generic variations and different strengths. Pharmacy phone numbers were used as the primary link because pharmacy IDs were unstable during ownership changes, while phone numbers remained constant. Patient matching was performed upstream by the EHR and commercial pharmacy data networks using vendor-specific proprietary matching algorithms and patient identity reconciliation processes. These matching procedures, which may incorporate multiple patient identifiers and vendor-defined heuristics, were not part of the custom code developed for this study and were not accessible to the study team. The study’s code used the EHR-assigned patient primary key to identify and filter orders associated with each patient.

Our approach was as follows: First, all antibiotic orders for AOM associated with relevant office visits and emergency department encounters were extracted, as described in step 3. Second, patient, medication, pharmacy, and provider information were joined to the order data. Third, using the outside pharmacy dispense data, the orders were joined using the internal EHR patient identifier, pharmacy phone number, or pharmacy ID. Fourth, the order medication GCN was matched to the prescription National Drug Code (NDC), and then to the GCN corresponding to that NDC. Finally, the prescription dispense date was matched to a date up to 14 days after the order date. Fourteen days was selected for our particular use case because AOM is expected to resolve within 2 weeks [20], and any symptoms persisting beyond this period are typically indicative of a new infection [21]. Readers may choose to change this dispense period depending on the type of behavior they expect from patients for the particular medication under study.

These joins are representative of the types of foreign keys needed to accurately join EHR order information to external prescription dispense data. We chose to use GCN, as it allowed for matches between brand and generic products. The pharmacy phone number was necessary because some pharmacies had changed ownership and the pharmacy ID in our EHR did not match the pharmacy ID in the dispense data, while the phone number remained the same.

In our logic, we did not use the prescriber name or NPI due to the heavy presence of trainees in our clinics, which means that the prescriber might not match the attending provider assigned to the encounter. Using the NPI could have been used to further restrict matching to the ordering provider in clinics that do not have such a large trainee presence. Potential joins are outlined in Figure 1, with details for consideration provided in Table 2.

To ensure that we had dispense data for each encounter, we limited the analysis to patients who had a dispense data triggering event at UCSF within 365 days of the initial encounter. Triggering events in our EHR were defined as follows: refill encounters, order encounters, hospital encounters, appointments, patient messages, or telephone encounters. This restriction was necessary as dispense data are retrieved from the pharmacy server only when the EHR identifies a triggering event. For scheduled appointments, this retrieval occurs via a batch job run during off-hours. This allows the data to be viewable to the outpatient clinician at the time of the appointment.

What constitutes a triggering event and the lookback period for retrieving dispense data will vary depending on an institution’s EHR configuration. At UCSF, our configuration’s look-back period pulled 365 days of dispense data for a triggering event. Once retrieval is initiated by a triggering event, the dispense history is extracted to the reporting server for future analysis during the standard extract, transfer, and load process for the EHR.

**Figure 1.**
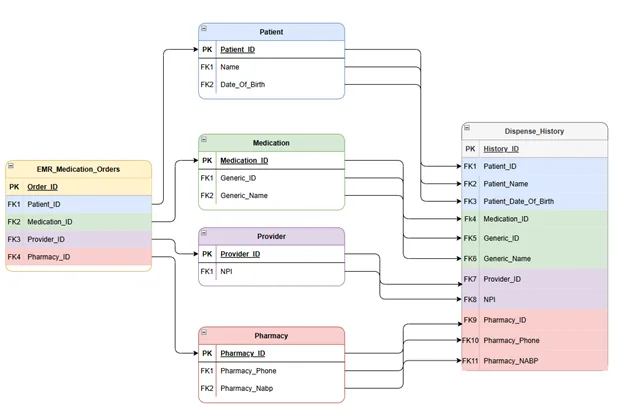
Conceptual flow diagram illustrating how patient, prescription, and pharmacy data could be linked in an SQL database to retrieve dispense data depending on researchers’ needs.

**Table 2.** Approaches for linking SQL tables to retrieve medication dispense data.

Concept	Option	Considerations
Patient	EHR^a^ patient identifier	Received data will already be linked to the patient—can only be used for integrated pharmacies. Users can consider birth date and full name if the patient ID is unavailable.
EHR order	EHR medication order ID	Can typically only be used for integrated pharmacies, as the order number will vary between in-house EHR and pharmacy databases.
Pharmacy	EHR pharmacy identifier, phone number, or NABP^b^	EHR pharmacy identifier may not be included in the outside data or could have duplicates if the pharmacy has changed ownership. Including the phone number or address may also be possible.
Prescriber	NPI^c^ and DEA^d^	Prescriber Name and NPI can be used to tie back to original order. Users should note that medication adjustments sometimes result in a change in the prescriber information sent back by the pharmacy. This is a strong consideration in trainee clinics, where attendings may change orders sent by residents.
Medication	EHR medication ID, generic identifier, generic name	EHR medication ID can exclude brand-generic matches. GCN^e^ or GPI^f^ are examples of generic identifiers that can be used. Additionally, matches across strengths are needed using the generic name will allow for this match.

a EHR: electronic health record.

b NABP: National Association of Boards of Pharmacy.

c NPI: National Provider Identifier.

d DEA: Drug Enforcement Administration.

e GCN: Generic Control Number.

f GPI: Generic Product Identifier.

### Step 5: Deduplication Across Data Sources

#### Case Study: Deduplication of Pharmacy Dispense Data

When dispense data are aggregated from multiple vendors, duplicate records must be identified and merged to avoid overcounting fulfillment. Multiple triggering events may pull the same medication dispense record. For example, consider a medication order for amoxicillin that is sent from an emergency department on a Monday and dispensed at an outpatient pharmacy later that same day. If you were to check the EHR that evening, you would not see the dispense because the patient would not yet have had a triggering event to bring that dispense from the pharmacy database into the institutional EHR. Imagine that later that same week, on Tuesday, the patient visits their physician for a different medication refill. At that Tuesday visit, the dispense from Monday would be evident because, in anticipation of the Tuesday appointment, the extract, transfer, and load would have run the night prior, retrieved the pharmacy database information, and incorporated it into the EHR.

However, most studies do not focus on such recent prescriptions but instead analyze long retrospective time periods. Going back to the example, imagine the patient then sees another provider on Thursday. If each of those encounters is a triggering event, the same dispense information would be retrieved on Tuesday and again on Thursday, leading to duplicate rows in the EHR.

This deduplication challenge arises because each triggering event returns the same underlying dispense record. In this example, the dispense that occurred on Monday, with triggering events on Tuesday and Thursday, would produce rows with identical dispense date, medication identifier, pharmacy identifier, and patient identifier. Without deduplication, these would appear as multiple dispenses. The researcher conducting a retrospective study must, therefore, distinguish data retrieved across multiple triggering events that may represent the same underlying dispense. Failing to do so could lead to grossly overestimating the number of dispenses, misrepresenting the true time of the dispense, or associating the wrong dispense with the encounter. In this example, it could appear that the patient received the prescription multiple times rather than once.

To address this, medication dispense data should be deduplicated by grouping key fields such as patient, dispense date, pharmacy, provider, and medication. This grouping produces a single record per dispense, regardless of whether the source data originated from a prescription-processing system or a payer claim.

Finally, your EHR may integrate data from multiple third-party dispense vendors. Depending on system configuration, one or more vendors may be queried per triggering event. The same grouping logic ensures a single-row representation even when multiple vendor sources contribute overlapping data.

#### Case Study Example

Of the 2 commercial pharmacy databases integrated into the EHR system, DrFirst provided the majority of the data, with 2345 (68.9%) exclusive entries, while SureScripts contributed 57 (1.7%) entries. Only 214 (6.3%) orders had dispense data available from both databases (Table 3).

This distribution is consistent with DrFirst serving as the preferred vendor within our system. Instances of overlap likely arise from multiple triggering events for a given prescription or from differences in data availability over time.

For example, dispense data may have been unavailable from DrFirst at an initial triggering event and was therefore pulled from Surescripts, but subsequently became available at a later event, resulting in retrieval from both sources across consecutive time points. In these instances, records were merged using the above deduplication grouping into a single “Dispensed” status to ensure that the match proportion was not artificially inflated.

**Table 3.** Commercial pharmacy database source (N=3404).

Database	n^a^ (%)
DrFirst only	2345 (68.9)
No dispense data	788 (23.1)
Both	214 (6.3)
Surescripts only	57 (1.7)

a Percentages are calculated from the total N and may not sum to 100% due to rounding.

### Step 6: Pharmacy Normalization

#### Case Study: Pharmacy Identifier Normalization

Pharmacy identifiers must be harmonized to account for discrepancies between the EHR’s “intended” sent to pharmacy and the “actual” dispensing pharmacy. Pharmacy addresses, phone numbers, National Association of Boards of Pharmacy (NABP) numbers, or NPI numbers can be used to match the dispense data to the order data. The pharmacy phone number and address often remain unchanged when a pharmacy changes ownership, whereas the NPI or NABP numbers are sometimes updated.

Additionally, it is helpful to categorize your pharmacy data into large groups based on the company. This becomes important during the validation step to determine whether pharmacies are sending dispense data to the database.

#### Case Study Example

We categorized 307 pharmacies into the following groups: CVS, Walgreens, Safeway, Rite Aid, Walmart, Costco, On-Site Hospital Pharmacy, Other, and Kaiser. Normalization revealed significant variation in data capture: large chains such as Walgreens and CVS showed an 82.4% and 81.9% match proportion, respectively, while “Other” pharmacies dropped to 49.3%, and Kaiser/Costco showed the lowest retrieval proportion (Table 4).

**Table 4.** Pharmacy locations, number of prescriptions sent, and number of prescriptions with recorded dispense data from our single academic institution^a^.

Pharmacy (n=307 locations)	Number of prescriptions sent (percentage of all prescriptions sent), n (%)^b^	Prescriptions dispensed (percent dispensed within this pharmacy chain), n (%)^b^
Walgreens (n=114)	1579 (46.4)	1301 (82.4)
On-site pharmacy (n=2)	994 (29.2)	671 (67.5)
CVS (n=96)	547 (16.1)	448 (81.9)
Safeway (n=29)	110 (3.2)	80 (72.7)
Other (n=30)	71 (2.1)	35 (49.3)
Rite Aid (n=16)	58 (1.7)	51 (87.9)
Walmart (n=10)	31 (0.9)	27 (87.1)
Costco (n=6)	9 (0.3)	2 (22.2)
Kaiser (n=4)	5 (0.1)	1 (20.0)
Total	3404 (100)	2616 (76.9)

a “Number of prescriptions sent” represents the number and proportion of prescriptions in the study cohort sent to each pharmacy. “Prescriptions dispensed” represents the absolute number of prescriptions that were dispensed and the respective proportion of prescriptions sent to that pharmacy that were ultimately recorded as dispensed in the EHR. These data are reported per encounter, not per patient; patients may have contributed multiple encounters during the study period.

b Percentages in the n (%) column are calculated from the total N and may not sum to 100% due to rounding.

### Step 7: Validation and Quality Assurance

#### Case Study: Linkage Validation and Pharmacy Reporting

Validation of the joins and pharmacy inclusion in the dispense data source is essential. First, evaluate the matched order-to-prescription data for a subset of your total data to validate that it is correctly using the logic to match the order to the dispense or claim. Second, gather the distinct pharmacies included in your order dataset and evaluate whether dispense data have been received for any patients. We also recommend comparing characteristics of linked vs unlinked orders (eg, patient demographics, encounter type, or pharmacy type) to assess potential bias in linkage.

To further ensure completeness of pharmacy reporting, we extracted the full list of pharmacies present in our order dataset and queried our EHR reporting database (Clarity) to determine whether each pharmacy had returned any dispense records for any medication and any patient during the study period. This step was used to confirm that pharmacies included in the analysis were actively transmitting data through the commercial pharmacy aggregation system into the EHR, rather than appearing not to have dispenses that were attributable to linkage or data availability issues. Specifically, we recommend pulling the first dispense date, last dispense date, and total number of dispenses for each pharmacy. This step confirms whether a pharmacy is a “reporting” pharmacy.

It is important to keep in mind that patients without a follow-up triggering event will not have any dispense data—which is why it is important to ensure that all included pharmacies are reporting. Generally, patients without a follow-up triggering event should be excluded from the analysis, as they will never have corresponding dispense data.

As an additional validation step, a subset of linkages could be reviewed to confirm concordance between ordered medications and dispensed products (eg, agreement with NDC). More comprehensive validation of linkage accuracy and data completeness—such as quantifying potential data transmission loss between the EHR and PDS—would require access to both systems. Such analyses were beyond the scope of this study but represent an important area for future work.

#### Case Study Example

Overall, 307 pharmacies were included in our cohort. Of these, 302 (98.3%) were confirmed to have returned at least 1 dispense record for any medication and for any patient in the EHR during the study period, indicating active data transmission. Within our study cohort, most patients elected to send their medications to large pharmacy chains such as CVS and Walgreens, whereas 994 (29.2%) prescriptions were sent to the local hospital pharmacy (Table 4). Only 71 (2.1%) encounters involved a pharmacy that was neither a major chain nor the local hospital pharmacy.

Of the 3404 antibiotic orders, 2616 (76.9%) were successfully linked to dispense data using the multikey strategy. Depending on which keys are used, this number will likely fluctuate. Linkage performance should also be evaluated under different combinations of available data elements, as missing or inconsistently captured fields may reduce the match proportion. To assess linkage validity, a subset of matched records was manually reviewed to confirm concordance between the physician notes, ordered medications, and dispensed products (eg, agreement between medication identity and the NDC).

In our dataset, on-site pharmacies demonstrated a lower observed dispense percentage. This likely reflects temporal operational factors during the study period, including a transition from a third-party vendor (Walgreens) to an internally managed UCSF pharmacy system, as well as concurrent contractual and data integration changes. These disruptions may have affected the capture and availability of dispense data in the EHR, leading to under-ascertainment of dispenses rather than true differences in patient medication pickup behavior.

## Discussion

### Principal Findings

This study demonstrates a methodology for using commercial pharmacy databases integrated into the EHR to assess medication adherence patterns, offering an alternative to traditional claims data. We obtained dispense data for 76.9% of orders for AOM. Given that more than 98.3% of pharmacies in our cohort have historically reported dispense data to our EHR, it is likely that the remaining 23.1% of orders were truly not dispensed rather than representing missing data.

The dispense proportion for antibiotics is consistent with prior reports for pediatric prescription dispense proportions—approximately 80% for pediatric pneumonia antibiotics [22] and 84% for pediatric antibiotics overall [23]—and is slightly higher than the 65% dispense proportion reported following emergency department visits [24]. It is important to note, however, that these figures reflect prescription dispensing rather than actual medication use; adherence to actually taking antibiotics is estimated to be closer to 62% [25]. In our cohort, DrFirst captured the majority of dispensed orders (n=2345, 68.9%), reflective of institutional agreement with it as our primary dispense source.

### Evaluating Data Key Logic

The choice of foreign keys for linking medications warrants careful consideration, as the selected medication identifier directly affects the logic used to match EHR orders to dispense records. Table 2 provides examples of the join granularity when different keys are used. Figure 1 illustrates example matches between data tables. Depending on the research question, some or all of these data elements can serve as keys, and the selection should align with the desired level of match. In addition to medication-specific keys, the other keys listed in the table represent options that can be leveraged depending on what is available in the dispense data at a given institution. For example, if patient ID is not present in the dispense history, secondary keys like patient name and date of birth may need to be used.

Using the provider as a matching key can be advantageous when the goal is to link dispenses to a specific prescriber. However, we did not use this approach in our study due to the complexity introduced by trainees such as residents and fellows. In these cases, a trainee may submit an order electronically, but if the pharmacy later calls to clarify formulation, quantity, or strength, the provider who responds may be different from the original order. The new prescription from the pharmacy will list the provider who answered the phone, which may differ from the encounter record. This issue is likely less problematic in settings without trainees and could be valuable when assessing dispense proportions at the provider level or when examining differences by specialty. Additionally, as our study restricted the date range for the dispenses, the chance of an additional outside prescriber writing the same medication in the dispense window was minimal.

We suggest that users consider the inclusion of the generic name or generic name ID, as it allows for the identification of generic substitutions and formulation changes, which are common in pediatric prescribing practices. For example, children may be prescribed a liquid but may arrive at the pharmacy, and their parents may request a pill if they have learned how to swallow them, or vice versa. They may need adjustments to the strength or formulation of amoxicillin-clavulanate depending on what the pharmacy has in stock. For any study that uses liquid medications or involves pediatric populations, we recommend keeping this generic identifier. This flexibility in prescribing may be less relevant in certain adult specialties, where medications may only be available in a single formulation.

While not the focus of this particular paper, it is worth mentioning that for institutions that connect to third-party vendors, these dispense data are often readily available to users, which allows clinicians to use these data in clinical practice. In the ambulatory setting, these data may be available under a medication history dispense tab and help providers identify the most recent antibiotic prescriptions or determine whether patients are picking up medications for a chronic condition, such as asthma inhalers or antidepressants. By integrating these data into routine workflows, clinicians can make more informed decisions about patient care, address potential barriers to adherence, tailor education during the visit, and then use the above-detailed methodology for large-scale epidemiological research. Excitingly, these data also have the potential to be used for clinical decision support tools, for example, by alerting clinicians when a patient appears to be nonadherent to their prescribed medications, enabling timely intervention [26].

### Limitations

The major challenge of this project was the lack of a distinct foreign key to directly link EHR prescribing data with pharmacy dispensing data. This limitation necessitated the use of multiple joins and matching variables, which may have introduced some degree of error into the dataset. While the matching process was carefully designed to minimize inaccuracies, the absence of a definitive linking mechanism remains a potential source of error. Readers should be especially cognizant of this step when recreating this tutorial for their own datasets.

Understanding which pharmacies are predominantly used by the patient population is critical when designing studies that rely on this type of data. Although this limitation was less relevant for our geographically concentrated pediatric population in the San Francisco Bay Area—where 98.3% of pharmacies reported data to the health information exchange—it remains an important consideration for other settings. Investigators should confirm that the dispense data in their EHRs adequately capture the population of interest to ensure the validity of their findings.

Restricting the cohort to patients who returned to UCSF within 365 days was necessary to enable querying of the pharmacy database server. While this approach ensured that dispense data would be captured when available, it introduced selection bias by excluding patients without follow-up, who may differ systematically in care access or utilization. This is a key limitation of the underlying data and technology. As a result, the method is less applicable to patients with only a single point of care, such as those seen in the emergency department. The approach is therefore most useful as a QI tool for patients with ongoing care at the site, whether through a primary care provider or a specialty clinic managing chronic conditions, such as rheumatology.

We limited the dataset to 14 days, which may have excluded additional dispenses that occurred later. For our purposes—focusing on an acute infection—a short timeframe was necessary. For studies of chronic conditions, where timing is less critical, this window could be extended to capture a more comprehensive medication use.

It is also important to note that, like claims data, dispense data do not equate to medication use. While the study successfully identified whether prescriptions were dispensed, it could not confirm whether patients adhered to the prescribed regimen or completed the course of antibiotics.

### Conclusions

Leveraging external pharmacy prescription dispense data integrated into the EHR offers a practical, timely method to study prescription dispense behavior at scale. Our experience linking pediatric AOM prescriptions to pharmacy databases demonstrates a dispense proportion of 76.9%, providing granular insights into antibiotic and pharmacy use. Although limitations remain—such as the absence of a definitive linking key and the need for patients to revisit the health care system—this tutorial underscores how SQL queries and careful matching processes can build a robust dataset. The approach is readily adaptable for diverse clinical applications, from acute infections to chronic conditions, and allows providers and researchers to more accurately gauge adherence during QI efforts.

## Supplementary material

10.2196/90954 Multimedia Appendix 1 Attrition diagram for encounter selection.

10.2196/90954 Multimedia Appendix 2 Distribution of unique patients and total encounters by number of encounters per patient (2930 patients; 3404 total encounters).

## Abbreviations

AOM: acute otitis media
EHR: electronic health record
GCN: Generic Control Number
GPI: Generic Product Identifier
ICD: International Classification of Diseases
NABP: National Association of Boards of Pharmacy
NDC: National Drug Code
NPI: National Provider Identifier
QI: quality improvement
SNAP: safety net antibiotic prescription
UCSF: University of California, San Francisco
