# Supplementary material for: Linking Dispense Data to Electronic Health Orders: Tutorial for Querying Commercial Pharmacy Databases to Support Systemwide Quality Improvement
Source: J Med Internet Res. 2026 Oct 6;28:e90954. doi: 10.2196/90954 (PMC13641662; doi:10.2196/90954)
Supplement: Supplementary file 1 [file jmir-v28-e90954-s002.docx]

| **Number of encounters per patient** | **Number of unique patients** | **Total encounters** |
| --- | --- | --- |
| 1 | 2,562 | 2,562 |
| 2 | 282 | 564 |
| 3 | 70 | 210 |
| 4 | 13 | 52 |
| 5 | 2 | 10 |
| 6 | 1 | 6 |
| **Total** | **2,930** | **3,404** |

Appendix Table 1: Distribution of unique patients and total encounters by number of encounters per patient (2,930 patients; 3,404 total encounters).
